# Curbing Lipids: Impacts ON Cancer and Viral Infection

**DOI:** 10.3390/ijms20030644

**Published:** 2019-02-02

**Authors:** Anika Dutta, Neelam Sharma-Walia

**Affiliations:** Department of Microbiology and Immunology, Chicago Medical School, Rosalind Franklin University of Medicine and Science, 3333 Green Bay Road, North Chicago, IL 60064, USA; anika.dutta@my.rfums.org

**Keywords:** PPAR, statins, fibrates, cholesterol, viruses, cancer, fatty acids

## Abstract

Lipids play a fundamental role in maintaining normal function in healthy cells. Their functions include signaling, storing energy, and acting as the central structural component of cell membranes. Alteration of lipid metabolism is a prominent feature of cancer, as cancer cells must modify their metabolism to fulfill the demands of their accelerated proliferation rate. This aberrant lipid metabolism can affect cellular processes such as cell growth, survival, and migration. Besides the gene mutations, environmental factors, and inheritance, several infectious pathogens are also linked with human cancers worldwide. Tumor viruses are top on the list of infectious pathogens to cause human cancers. These viruses insert their own DNA (or RNA) into that of the host cell and affect host cellular processes such as cell growth, survival, and migration. Several of these cancer-causing viruses are reported to be reprogramming host cell lipid metabolism. The reliance of cancer cells and viruses on lipid metabolism suggests enzymes that can be used as therapeutic targets to exploit the addiction of infected diseased cells on lipids and abrogate tumor growth. This review focuses on normal lipid metabolism, lipid metabolic pathways and their reprogramming in human cancers and viral infection linked cancers and the potential anticancer drugs that target specific lipid metabolic enzymes. Here, we discuss statins and fibrates as drugs to intervene in disordered lipid pathways in cancer cells. Further insight into the dysregulated pathways in lipid metabolism can help create more effective anticancer therapies.

## 1. Introduction

### 1.1. Cancers and Infection Related Cancers

Cancer is a leading cause of death worldwide [1]. In 2018, 609,640 cancer deaths and 1,735,350 new cancer cases were projected to occur in the United States alone [2]. The most deaths are caused by breast, gastric, liver, lung, and colon cancer [1]. Lung cancer is the leading cause of cancer-related death worldwide and in the United States. Lung cancer is also the largest contributor to new cancer diagnoses [3]. Breast cancer is the second most common cancer in women and accounts for 25% of all cancer diagnoses in American women [4]. Gastric cancer is the second most commonly occurring cancer worldwide and the fourth and fifth most common cancer in men and women, respectively [1]. Colon cancer is the third most common cancer worldwide and its likelihood of diagnosis increases progressively from age 40 [5]. Lastly, liver cancer is the fifth most common cancer in the world and has a poor survival rate due to its aggressive nature [6].

Viruses are estimated to cause about 15% of all human cancers worldwide, and most of these tumor viruses are hooked on lipid signaling, synthesis, and metabolism [7]. DNA viruses that contribute to human cancers include human papillomavirus (HPV], Epstein–Barr virus (EBV), Kaposi’s sarcoma-associated herpesvirus (KSHV)—also known as human herpesvirus 8 (HHV-8), Merkel cell polyomavirus—a polyomavirus (MCPyV) associated with the development of Merkel cell carcinoma (MCC) and hepatitis B virus [7]. The two RNA viruses that can cause the development of human cancer are hepatitis C and human T lymphotropic virus (HTLV-1] [7]. EBV and KSHV are both herpesviruses with DNA genomes [7]. EBV is associated with Hodgkin’s disease, B and T cell lymphomas, post-transplant lymphoproliferative disease [8], nasopharyngeal carcinomas, and leiomyosarcomas [7]. It has been associated with up to 10% of all gastric cancers, and up to 200,000 new malignancies every year worldwide [9,10]. A vaccine to prevent or treat EBV has not yet been licensed [10]. KSHV is similar to EBV in that the B lymphocyte is the predominant infected cell, and it has been estimated to cause 34,000 new cancer cases globally [7,11]. It is the leading cause of AIDS-related malignancy and cancer mortality [12]. Kaposi’s sarcoma (KS] is the most common AIDS-defining cancer [13,14,15,16]. KS is a serious clinical problem prevailing in up to 50% of HIV+KS+ patients in the United States and 19–61% in Sub-Saharan Africa, who never regain remission even after combination of anti-retroviral therapy (cART] [17,18,19]. HPV is a DNA tumor virus that causes warts or benign papilloma, and persistent infection is associated with the development of cervical cancer [7]. It infects epithelial cells, integrates into host DNA, produces E6 and E7 oncoproteins, and disrupts tumor suppressor pathways to encourage the proliferation of cervical cancer cells [7]. It also plays a role in cancers of the skin, head, and neck [7]. The HPV vaccine is effective against HPV 16 and 18, but it does not protect against all high-risk HPV types and may not benefit women who are already infected [7]. 

Hepatitis C virus (HCV] and hepatitis B virus (HBV) together cause 80% of hepatocellular carcinoma cases [7]. Hepatitis C is an RNA virus that can infect liver cells and cause acute and chronic hepatitis [7]. Infection with hepatitis C virus can result in cirrhosis, which can then lead to primary hepatocellular carcinoma [7]. By contrast, hepatitis B is a DNA virus, but it can also cause acute and chronic hepatitis, which can lead to cirrhosis, liver failure, and hepatocellular carcinoma [7]. Both of these viruses could use new methods of treatment. Hepatitis C is not well suited to vaccines, because its genome mutates at a high rate and therefore it is able to escape elimination and immune recognition [7]. Hepatitis B does have a vaccine, but up to 10% are non-responders, and HBV infection still causes an estimated 1 million deaths annually [7,20]. HTLV-1 is an RNA retrovirus that has been linked to adult T-cell leukemia and a variety of chronic inflammatory diseases including [7]. Once tumor formation begins, progression is very rapid [7]. Chemotherapy for HTLV-1 associated adult T-cell leukemia can be at first beneficial but relapse is common and survival is an average of eight months [7]. 

There is a need for therapies that can block viral replication, and studies show that viral entry and release, and consequently replication, can potentially be blocked if membrane lipid composition is altered [21]. Additionally, modification of lipid metabolism may offer new possibilities for antiviral therapies [21].

### 1.2. Diet and Obesity in Cancer

**Figure 1:**
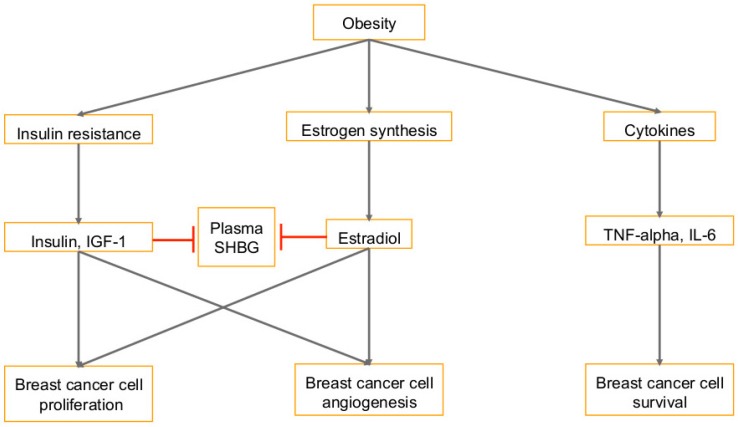
Pathways that link breast cancer with obesity: Several consequences of obesity, such as insulin resistance, higher levels of circulating estrogen, and secreted cytokines play a role in the development of breast cancer. Insulin and IGF-1 promote proliferation and angiogenesis by activating the PI3K/Akt and Ras/Raf/MAPK pathways. Insulin also inhibits sex-hormone-binding globulin (SHBG), which binds testosterone and estradiol so there is increased free estradiol. Circulating estrogens promote growth of breast epithelial cells and lead to more proliferation and angiogenesis as well. Adipocytes can secrete pro-inflammatory cytokines which stimulate more lipolysis and further release of free fatty acids to promote cancer cell survival. T-bars in red denote inhibition.

Nutrition, diet, obesity, hyperlipidemia, hyperglycemia, and other modifiable risk factors such as lack of exercise, hypertension, and insulin resistance have been linked to type 2 diabetes, cardiovascular disease, hypertension, and several cancers such as breast, endometrial, pancreatic, kidney, gallbladder, colorectal, and ovarian cancers [22,23]. Obesity is a risk factor for breast cancer [23,24]. Overweight and obese breast cancer patients have an increased risk of lymph node metastasis, large tumors, and mortality [24]. There are several hypotheses as to why obesity is correlated with breast cancer. (A) The first is that the increased amount of adipose tissue in obese women means more peripheral aromatization of androgens, which causes higher levels of circulating estrogens (Figure 1) [24]. (B) Another hypothesis (Figure 1) is that obesity leads to higher levels of circulating insulin and insulin-like growth factor (IGF), which act as mitogens [24]. Extra adipose tissue releases additional non-esterified fatty acids, which leads to the development of insulin resistance [24]. Tissues are not able to efficiently absorb glucose and so the pancreas increases insulin secretion in both fasted and fed states [24]. Insulin is necessary for cell growth and it promotes DNA synthesis [24]. It also increases levels of insulin-like growth factor 1 (IGF-1), which can target breast epithelial cell receptors and induce anti-apoptotic and mitogenic pathways [24]. Half of the breast tumors have been shown to overexpress the IGF-1 receptor, and inactivation of the receptor leads to diminished mammary tumor growth [24]. Insulin and IGF-1 promote angiogenesis, increase cell proliferation, and inhibit apoptosis [25]. 

Estrogen and the insulin/IGF-1 pathway work together (Figure 1) in breast epithelial cells to increase transcriptional activation of estrogen receptor (ER-α) and induce mitogenic responses [24]. Estrogen does this by stimulating resting breast epithelial cells in G0/G1 to re-enter the cell cycle and go through cell division [24]. This is mediated by c-Myc, a transcription factor that is induced with estrogen stimulation [24]. With higher levels of insulin and IGF-1, concentrations of sex-hormone binding globulin (SHBG) are reduced [24]. SHBG binds estradiol and testosterone, so a decrease in its levels leads to an increase in circulating estradiol [24]. SHBG binds to breast cancer cells to inhibit estradiol-induced cell proliferation, and incubation of breast cancer cells with SHBG before treatment with estradiol cancels out the anti-apoptotic effect of estradiol [24]. It has been shown that breast cancer risk is inversely correlated with blood levels of SHBG (Figure 1) [24]. (C) The third hypothesis is that adipocytes are like endocrine cells that secrete hormone-like molecules and cytokines [24]. When invasive tumors penetrate the basement membrane and tissue barriers, the adipocytes and breast cancer cells can simply participate in paracrine interactions [24]. Studies have shown that tumor growth can be directly influenced by adipose tissue; mice injected with adipose tissue and mammary carcinoma cell line SP1 developed tumors, but no tumor growth was observed with injection of SP1 far from any fat [24]. Furthermore, breast cancer cells that were treated with adipocyte-conditioned media upregulated proliferation and metastasis while also downregulating BRCA1-associated RING domain protein 1 (BARD1), a tumor suppressor, and p18, a cell-cycle checkpoint inhibitor [24]. Breast cancer tumors injected with adipocytes grew to be three times as large as the tumors injected with fibroblasts [24].

Adipocytes secrete tumor necrosis factor-alpha (TNFα), an inflammatory cytokine [24]. Its expression is increased in obese rodent models and it inhibits the insulin receptor signaling pathway, thus assisting in the development of insulin resistance [24] (Figure 1). Adipocytes also secrete IL-6, high levels of which are associated with poor prognosis in breast cancer [24]. IL-6 production is also associated with the signals from prostaglandin PGE_2_, which induces DNA transcription for IL-6 synthesis [26,27,28] (Figure 1). IL-6 activates the mitogen-activated protein kinase (MAPK) pathway which promotes cell migration, and it inhibits the activation of proteases that are involved in apoptosis [24]. IL-6 also inhibits cell differentiation and promotes osteoclast formation and therefore promotes metastatic growth [24]. In a previous study from our lab, we imaged three-dimensional (3D) sphere cultures of primary human mammary epithelial cells (HMEC), highly invasive breast cancer (SUM1315MO2) and primary inflammatory breast cancer (SUM149PT and SUM190PT) cells [29]. We found that the SUM1315MO2 and SUM149PT spheres were larger with differences in morphology, composition and their microenvironment [29]. We performed cytokine profiling of HMEC, SUM1315MO2 and SUM149PT conditioned media, which showed an abundance of inflammatory cytokines and chemokines such as interleukins IL-6, IL-8, and IL-17. Levels of survival kinases such as AKT, p44/42 MAPK, p65, and GSK3β were also, higher in breast cancer SUM1315MO2 and SUM149PT spheres when compared to HMEC spheres [29]. Our study [29] for the first time, revealed that osteoprotegerin (OPG) is secreted and expressed at very high levels from the SUM1315MO2 invasive breast cancer cell line, as well as the SUM149PT and SUM190PT inflammatory breast cancer cell lines when compared to healthy HMECs. Our study [29] demonstrated specific OPG staining in inflammatory breast cancer patient tumor sections. Interestingly, immunoprecipitation of breast cancer cell extracts by OPG antibody revealed lipid metabolic enzyme, fatty acid synthase (FASN), which is a key enzyme of the fatty acid biosynthetic pathway [30,31]. FASN controls the process of producing de novo fatty acids from carbohydrate and amino acid-derived carbon sources [32]. Adult body mass index (BMI) is a reflection of the accumulation of adipose tissue [33]. Obesity has also been linked with gastric cancer and its complications including gastroesophageal reflux, insulin resistance, high adiponectin, leptin, and an abnormally high blood level of IGF [34] (Figure 1). 

## 2. Lipid Synthesis in Human Cancers and Viral Infection Linked Cancers

### 2.1. Regulation of Lipids in Membrane Microdomains

The goal of lipid synthesis is to convert carbons derived from nutrients into fatty acids, cholesterol, phosphoglycerides, eicosanoids, and sphingolipids [35,36]. Cancerous cells show an increased rate of lipid synthesis, which has several important functions [35,36]. Lipid (fatty acids) compositional complexity, versatility, repertoire, fluidity, and lipid asymmetry is very essential to determine the characteristics of the membrane, rafts or even cell per se [35,36,37]. The membranes include the organelles such as the mitochondria, Golgi and the endoplasmic reticulum [35,36,37]. Therefore, changing lipid properties can drastically affect biomembranes, their topology, spatial organization and overall cellular machinery [35,37]. Higher levels of lipid saturation in the cell membrane protect cancer cells from oxidative damage by reducing oxidative degradation of lipids and may inhibit chemotherapeutic drug uptake [35,36,38]. Breast cancer cells have less membrane fluidity because of the increased levels of lipids, and inhibition of their synthesis is associated with apoptosis and cell cycle inhibition [35,36,37,38]. 

Lipids also function as signaling molecules in cancer [35]. Phosphoinositides are lipid second messengers that relay signals to the cellular machinery from activated growth factor receptors [35,39]. Lysophosphatidic acid (LPA) is another lipid second messenger that binds to G-protein-coupled receptors and activates cell migration, proliferation, and survival [35,40]. Ceramides, which are involved in inducing apoptosis and arresting cell growth, are downregulated in cancer cells [35,41,42]. Conversely, sphingosine-1-phosphate (S1P), which promotes angiogenesis and cell growth, is upregulated in cancer cells [35,43,44,45,46]. Eicosanoids regulate inflammation and thus assist in tumor progression [35,47,48]. Lipids function in protein regulation as well [35]. Prenylation facilitates the activity and localization of several signaling proteins [35,49,50]. Glycosylphosphatidylinositol (GPI) targets proteins to the outer layer of the plasma membrane [35]. Protein trafficking and localization requires different kinds of lipid anchors [35]. Association with membrane rafts is promoted by protein modification with saturated acyl chains [35]. In contrast, unsaturated fatty acids keep proteins out of cholesterol-rich membrane rafts. Regulation of growth factors is also associated with protein acylation [35,51]. Lastly, lipids are associated with autophagy—a self-destructive mechanism required under nutrient-poor conditions to remove dysfunctional components [35,52,53,54]. This allows for cancer cells to conserve their energy during nutrient limitation and therefore promotes cell survival [35]. The overexpression of lipid metabolism-related genes such as ATP-binding cassette transporter (*ABCA1*), acyl-coA synthetase long-chain family member 1 (*ACSL1*), 1-acylglycerol-3-phosphate *O*-acyltransferase 1 (*AGPAT1*) and stearoyl-CoA desaturase (Δ-9-desaturase) (*SCD*) has been proposed as a prognostic marker of stage II colorectal cancer (CRC) and is also called a ColoLipidGene signature [55]. Rectal adenocarcinoma (RAC), a common malignant tumor of the digestive tract is also linked to lipid peroxidation related oxidative stress, and plasmalogen alterations [56]. Pancreatic ductal adenocarcinoma (PDAC), a devastating disease is related to the intake of total fat, but especially of saturated and mono-unsaturated fatty acids (MUFAs) [57]. Given the functions of lipids in membrane structure, cell signaling, and post-translational modification of proteins, it is clear that lipids have vital roles that regulate the survival and proliferation of cancer cells [35,58]. We will focus primarily on aberrant cholesterol and fatty acid synthesis.

Lipids play an important role in viral infection, as they are the structural elements of cellular and viral membranes [21]. Viruses target lipid synthesis and signaling to remodel their host cells and generate lipids for the viral envelope [59]. Lipid interactions such as membrane fusion, envelopment, and remodeling are vital for viral replication, and compounds that affect lipids such as cholesterol and sphingolipids interfere with viral replication [21]. Viruses replicate inside the host cell, so they have to cross the host cell membrane for entry and exit [21]. Lipids have several roles in viral entry. They can function as direct and indirect viral receptors, as entry cofactors, and fusion cofactors [59]. Lipids are involved in viral replication in several ways. They have a role in phosphoinositide signaling to reorganize the membrane or bind viral proteins [59]. Viruses may also generate lipids at sites of replication by promoting lipid biosynthesis [59]. By inducing lipid metabolism, viruses exploit the energy in lipid stores during their replication [59]. Viruses can induce autophagy to degrade lipid droplets and release lipids, which are oxidized in mitochondria to generate ATP [59]. In addition to providing energy, lipid droplets can aid in viral assembly and budding [59]. Lipids may also facilitate viral exit by use of the VLDL secretion machinery [59]. Knockdown of apolipoproteins ApoE and ApoB decreased the amount of secreted infectious virus [59].

Hepatitis C affects lipid metabolism and uses it to its advantage throughout the infectious cycle [60]. An increase in lipid droplets has been found in liver biopsies of infected patients [60]. EBV-encoded latent membrane protein 1 (LMP1) has been shown to promote cell proliferation and progression of nasopharyngeal carcinoma via activation of SREBP1-mediated lipogenesis [61]. Short-chain fatty acids (SCFA) stimulate the two related human gamma-herpesviruses to enter the lytic cycle through different pathways of chromatin remodeling [62]. EBV LMP1 has been shown to reorganize membrane lipid rafts and cytoskeleton microdomains to modulate phosphatidylinositol 3-kinase (PI3K) and its downstream target, Akt signal transduction [63]. 

In patients infected with human T lymphotropic virus (HTLV-1), significantly higher levels of VLDL and triglycerides were detected [64,65].It has also been found that disruption of lipid rafts can lead to a decrease in infection by HTLV-1 [66]. HTLV-1 encoded Tax1 protein has been associated with the accumulation of cellular alterations that promote leukemia in infected HTLV-1-infected individuals [66]. The cytoplasmic Tax1 protein persistently resides in the Golgi-associated lipid raft microdomains and Tax1 directs lipid raft translocation of IKK through selective interaction with IKKγ [67,68,69]. Depletion of IKKγ impairs Tax1-directed lipid raft recruitment of IKKα and IKKβ suggesting that Tax1 actively recruits IKK to the lipid raft microdomains for continuous/sustained NF-κB activation and contributes to HTLV-1 infection linked tumorigenesis [67,68,69]. 

### 2.2. Association of Lipid Pathways with Glycolysis, Fatty Acid Synthesis, and Glutaminolysis

High-throughput RNA sequencing demonstrated significant changes in genes involved in overlapping lipid-related functions and/or glucose metabolism disorder in KS lesions [70]. KSHV infection has been shown to induce glycolysis, glutaminolysis, and fatty acid synthesis pathways, for the survival of latently infected endothelial cells [71]. KSHV infection of primary endothelial cells utilizes the host lipid raft-dependent macropinocytosis pathway and endosomal sorting complexes required for transport (ESCRT)-0 proteins for entry [72,73]. KSHV ORF45, a viral protein in the tegument layer, connecting capsid and envelope, associates with lipid rafts of host cellular membrane triggering KSHV budding for final envelopment and virion maturation [74,75,76]. Lipid metabolism plays a vital part in KSHV infection and therefore may be used as a drug target [12,76]. KSHV can manipulate lipid biosynthesis in a host cell to promote viral infection and tumorigenesis in several ways [12,76]. Lipids play a role in the initial infection, survival and proliferation, reactivation, and angiogenesis of KSHV infected cells [12,76]. Studies have shown that lipids are important for the survival of KSHV-infected cells and that they have higher rates of fatty acid synthesis and aerobic glycolysis than primary B cells [12,76]. Reprogramming of cholesteryl ester metabolism has been demonstrated to be involved in regulating neo-angiogenesis and metastasis in KSHV infected endothelial cells [77]. KSHV stabilized hypoxia-inducible factors (HIFs) has been reported to play a critical role in KSHV latency, reactivation and metabolic reprogramming (carbohydrate, lipid, and amino acids) [78]. 

## 3. Cholesterol Synthesis in Human Cancers and Viral Infection Linked Cancers

Cholesterol has vital physiological roles such as controlling membrane fluidity and using cell signaling to regulate cell growth, proliferation, and migration [79]. It is also a precursor for steroid hormones which activate nuclear receptors to control inflammation and immune functions [79]. Cholesterol is transported from the liver to cells through the bloodstream in a low-density lipoprotein (LDL) bound form [80]. Cells take-up the LDL using clathrin mediated endocytosis and the endocytic pathway transports it to lysosomes where it is hydrolyzed to free cholesterol molecules [80]. The cholesterol molecules are then taken to the membrane-bound organelles and the cell membrane [80]. 

Cholesterol levels are tightly regulated in the body [79]. The key regulators are sterol regulatory element-binding protein transcription factor 2 (SREBF2) and liver x receptors (LXR; LXRα and LXRβ) [80]. Levels of endoplasmic reticulum (ER) cholesterol are used to sense for intracellular cholesterol homeostasis [80]. If there is a decrease in ER cholesterol, SREBF2 is translocated from the ER to Golgi to the nucleus to activate gene transcription for cholesterol synthesis [80]. Conversely, if there is an increase in cholesterol levels, its synthesis is shut down and its export is facilitated by activation of LXR receptors [80]. LXRs are sterol-sensitive transcription factors of the nuclear receptor superfamily. LXRs regulate the expression of several genes involved in the uptake, transport, efflux, and excretion of cholesterol in a tissue-dependent manner. These are also crucial regulators of the reverse cholesterol transport pathway and subsequently whole-body cholesterol content [81].

**Figure 2:**
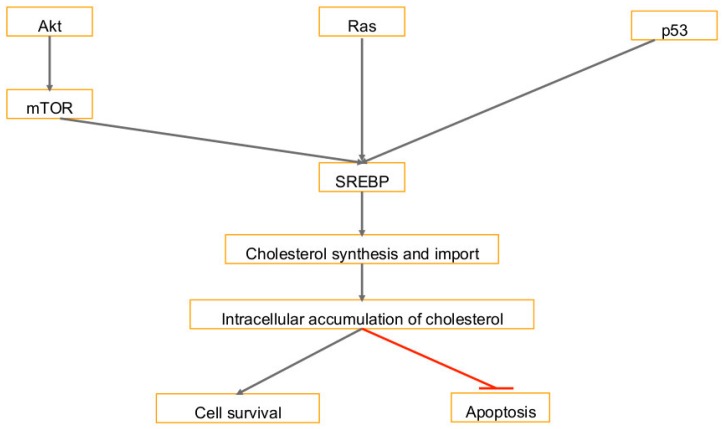
Pathways that lead to an accumulation of cholesterol: Activation of SREBP transcription factor induces cholesterol. This is induced by activation of PI3K/Akt/mTOR signaling, cancer gene RAS, and dysregulation by TP53. The accumulation of cholesterol in the cell promotes survival and inhibits apoptosis. T-bar in red denotes inhibition.

There are several signaling pathways that activate cholesterol synthesis in cancer cells [80]. Intracellular cholesterol levels are promoted by the activation of PI3K/AKT signaling, which induces cholesterol synthesis by activating the SREBP transcription factor, the regulator of cholesterol synthesis encoding genes [80] (Figure 2). SREBP is activated by inhibiting mTORC1 dependent ABCA1 mediated cholesterol export and activating LDL receptor-mediated cholesterol import pathway [80]. Promotion of cholesterol synthesis by the AKT/mTORC1/SREBP pathway contributes to cell growth, bone metastases, and cancer aggressiveness [80] (Figure 2).

Cholesterol synthesis is also activated through TP53, a frequently mutated gene in cancer [80]. In breast cancer, the cholesterol synthesis pathway is unregulated by the loss of TP53 function [80]. This disrupts the breast tissue architecture and induces proliferation [80]. In studies where the mutant TP53 was knocked down, the morphology of the breast cancer cells changed from the disorganized back to a normal phenotype [80] (Figure 2).

In cancer cells, high levels of mitochondrial cholesterol lead to resistance to apoptotic signals [80]. Cholesterol import into the mitochondria is regulated by the two proteins steroidogenic acute regulatory (STAR) and STAR-related lipid transfer domain containing 3 (STARD3) [80]. In human epidermal growth factor receptor 2 (HER2/neu, c-erbB2) positive breast cancer cells, STARD3 is associated with a poor prognosis, and lower levels of STARD3 increase cell death while reducing cell proliferation [80]. HER2 is a membrane tyrosine kinase and oncogene, when activated it provides the cell with potent proliferative and anti-apoptosis signals, and confers aggressiveness to breast cancers. Higher levels of STARD3 also decrease the adhesiveness of breast cancer cells, which promotes metastases [80]. Another gene that controls cholesterol homeostasis is ABCA1, a cell membrane cholesterol exporter, and it is dysregulated in cancer cells [80]. Lower levels of ABCA1 increase mitochondrial cholesterol levels and promote cancer cell survival [80]. Growing tumors have been found to have 3-fold lower levels of ABCA1 expression as opposed to normal cells [80]. 

High cholesterol is a risk factor for several pathologies and is associated with the development of cancer [79]. It has been observed that cholesterol promotes cell proliferation and migration [79]. It accelerates the formation of tumors, enhances tumor angiogenesis, and increases their aggressiveness [23]. Cholesterol is also associated with chemotherapy resistance [79]. In breast cancer tumors, higher plasma cholesterol levels are associated with higher expression of cyclin D1, an oncogenic driver [23]. Furthermore, high cholesterol content in lipid rafts is associated with higher rates of cell survival in prostate cancer cells [23]. Lipid rafts are implicated in Akt activation, which then phosphorylates pro-apoptotic proteins and inactivates them [82]. Cholesterol is a major component of cellular and mitochondrial membranes; the inhibition of cholesterol synthesis may inhibit the formation of new membranes demanded by proliferating tumor cells [83]. It was observed that although lipogenesis was upregulated in cancer cells, during tumor development, plasma cholesterol levels were reduced [23]. This suggests that transformed cells may utilize more cholesterol than normal cells; thus regulation of the cholesterol synthesis pathway may limit cellular proliferation [84]. Cholesterol metabolic pathways are required for the replication, secretion, and entry of HCV and drugs targeting cholesterol metabolic pathways have potential in treating HCV infection [85]. Cholesterol has also been identified as a critical factor for EBV latent membrane protein 2A trafficking and protein stability as it regulates LMP2 phosphorylation and ubiquitination [86]. Treatment of cells with methyl-beta-cyclodextrin (MβCD), which depletes cholesterol from the plasma membrane, increased LMP2A levels, its secretion in exosomes and blocked LMP2A endocytosis resulting in LMP2A abundance in the plasma membrane [86]. 

**Figure 3:**
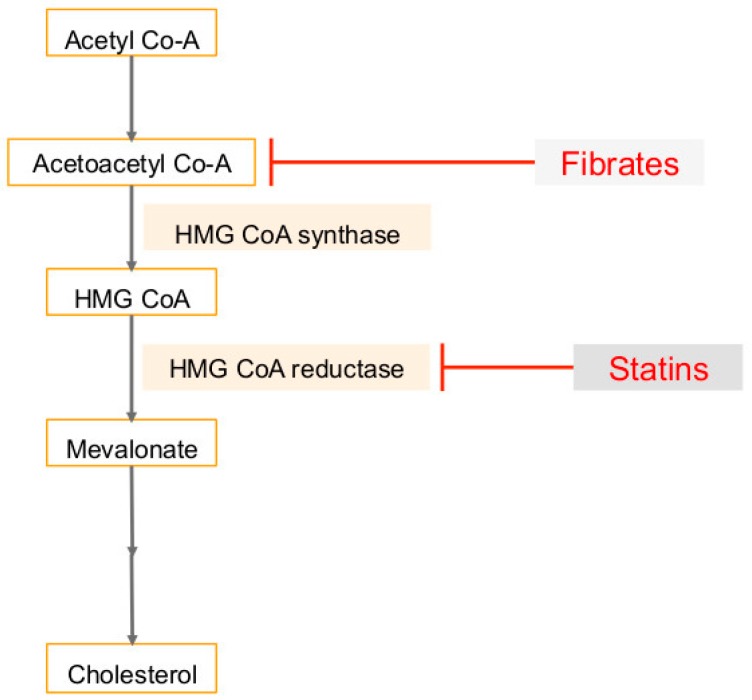
Mevalonate pathway as an important metabolic pathway: The mevalonate pathway is regulated by HMG CoA reductase (HMGCR), and this enzyme is targeted by statins to decrease plasma cholesterol. Fibrates target the mevalonate pathway by inhibiting acetoacetyl coenzyme A. This reverses the effects of cholesterol to inhibit cell proliferation and trigger apoptotic parameters. Downregulating the pathway also suppresses production of farnesyl pyrophosphate and geranylgeranyl phosphate to inhibit the invasive properties of cancer cells. T-bars in red denote inhibition.

The mevalonate (MVA) pathway, which leads to the production of cholesterol, can be dysregulated in tumor cells [87] (Figure 3). Many of the downstream products are required for protein synthesis, membrane integrity, signaling, and cell-cycle progression, and are therefore critical in cell proliferation [84]. Higher levels of enzymes in the MVA pathway are associated with rapid progression and poor prognosis in cancer patients, and treatment with mevalonate promotes proliferation of breast cancer cells and tumor growth [82,87] (Figure 3). This pathway is upregulated by mutated p53, a tumor suppressor protein [84]. The increased proliferation rates are associated with a faster entry of cells through the G1 restriction point and into S phase [88]. These cells have more activating phosphorylation of cyclin-dependent kinase-2 (CDK-2), which controls initiation of DNA synthesis and replication, and decreased inhibitory binding of CDK-2 to p21, a regulator of the G1 restriction point [88]. The disruption of this pathway in malignant cells may result in the inhibition of cell-cycle progression and reduce proliferation and metastasis of cancer cells [84].

Hepatitis C virus requires geranylgeranylation, a metabolite of the mevalonate pathway, to allow binding of viral protein NS5A to viral cofactor FBL2 [89,90]. In the mevalonate pathway, simvastatin interferes with the activity and localization of EBV latent membrane protein 1 LMP-1 to induce apoptosis [91]. KSHV viral microRNAs (miRNAs) have been shown to target 3-Hydroxy-3-methylglutaryl-coenzyme A (CoA) synthase 1 (HMGCS1), 3-hydroxy-3-methylglutaryl-CoA reductase, enzymes in the mevalonate/cholesterol pathway [89]. Addition of 25-hydroxycholesterol to primary cells inhibited KSHV infection suggesting that KSHV miRNAs decrease the level of 25-hydroxycholesterol and promote viral infection [89]. 

## 4. Fatty Acid Synthase (FASN) in Cancers and Viral Infection-Associated Cancers

**Figure 4:**
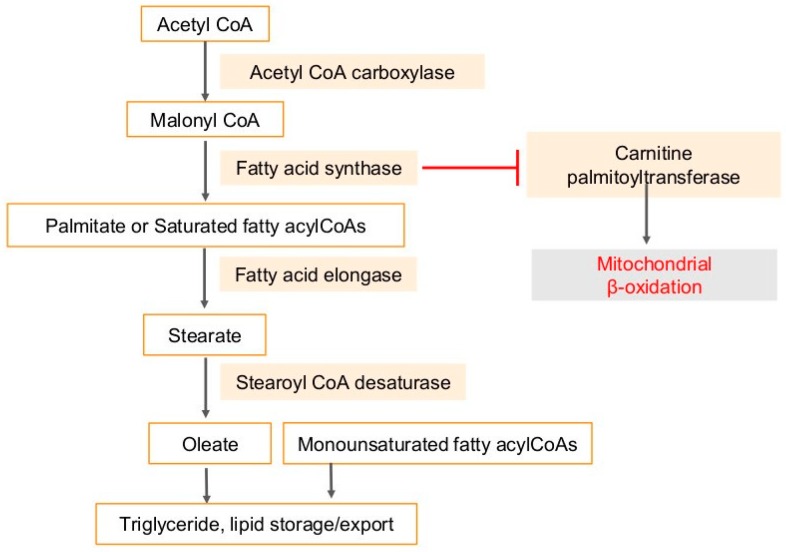
Triacylglycerols are synthesized from acetyl-CoA. Acetyl-coA is metabolized to malonyl-CoA via acetyl-CoA carboxylase, which in turn is converted to Palmitate, the principal product of the fatty acid synthase system in animal cells. Palmitate is lengthened to form stearate by enzyme fatty acid elongase. Stearate, a saturated fatty acid is subsequently metabolized by stearoyl-CoA desaturase enzyme, that forms a double bond in stearoyl-CoA, leading to the monounsaturated fatty acid oleic acid. Carnitine palmitoyltransferase (CPT), the enzyme in the outer mitochondrial membrane, converts long-chain acyl-CoA species to their corresponding long-chain acyl-carnitines for transport into the mitochondria. CPT induces mitochondrial β-oxidation, which is a complex pathway involving energy metabolism. FASN inhibits CPT with resultant inhibition of fatty acid oxidation. T-bar in red denotes inhibition.

Besides cholesterol, triacylglycerol can also be synthesized from acetyl-CoA [92] (Figure 4). Malonyl-CoA is produced from acetyl-CoA via acetyl-CoA carboxylase (ACC), which is converted to Palmitate. Palmitate is lengthened to form stearate by enzyme fatty acid elongase (Elovl #1–7) [92] (Figure 4). Fatty acid elongation primarily occurs in the endoplasmic reticulum and uses malonyl-CoA and fatty acyl-CoA as substrates. Another endoplasmic reticulum-bound enzyme is stearoyl-CoA desaturase (SCD), which catalyzes the desaturation of saturated palmitoyl- and stearoyl-CoA, those are converted to palmitoleoyl- and oleoyl-CoA, respectively [92] (Figure 4). Multiple drugs targeting lipid pathways are in clinical trials. Antitumor synergy has been observed in vitro and in vivo combining A939572 with an mTOR inhibitor in ccRCC and A939572 is a small molecule that specifically inhibits SCD1 enzymatic activity [93,94,95]. Similarly, T-3764518, a novel and orally available small molecule inhibitor of SCD1 showed promising antitumor effects in colorectal cancer HCT-116 cells and their growth and mesothelioma [96,97].

**Figure 5:**
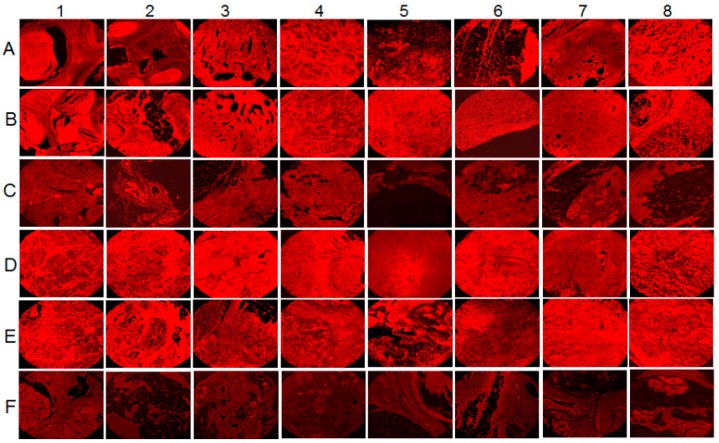
Fatty acid synthase (FASN) levels in human breast cancer tissue samples. 16 breast cancer tissue samples, in duplicates (**A**,**B**,**D**,**E**) along with their controls (**C**,**F**) were analyzed by IHC staining for FASN. Magnification for the panels is 4×.

FASN, the enzyme responsible for de novo fatty acid synthesis, is expressed at higher levels in breast, prostate, colon, and ovarian cancer cells as opposed to normal human mammary epithelial cells (HMEC) [98]. We analyzed the breast tissue sections of healthy subjects and breast cancer patients for the presence of FASN by immunofluorescence staining using anti-FASN antibody (unpublished results). Abundant FASN expression was detected in breast cancer tissue sections (Figure 5A,B,D,E) compared to the normal healthy control tissue sections (Figure 5C,F). We next evaluated the fold change in FASN expression in all 32 sections by densitometry analysis using ImageJ software. A 0–2, 2–4, 4–6-fold induction in FASN expression was observed in 14, 12 and 6 tumor sections, respectively. Collectively, these results highlight the presence of PPARα expression in human breast cancer tissues.

Our previous studies show larger numbers of lipid droplets in SUM1315MO2 (invasive breast cancer cell line) and SUM149PT cells (inflammatory breast cancer cell lines) when compared to HMEC using electron microscopy, immunofluorescence, and fluorescence quantitation [30]. SUM1315MO2 and SUM149PT also had higher expression of FASN when compared to HMEC using immunoblotting and immunofluorescence staining [30]. In addition, Western blot analysis showed higher levels of cyclooxygenase-2 (COX2), which is important in cancer progression and inflammation, in SUM1315MO2 and SUM149PT cells [30]. To investigate the importance of FASN and COX2 in cancer cell progression, these two enzymes were blocked with C75 (FASN inhibitor) and/or celecoxib (COX2 inhibitor) and the results showed a reduction in the amount of lipids formed per cell as well as decreased levels of cell survival proteins p-Erk and p-GSK3β [30]. Since high levels of FASN leads to more lipogenesis, which causes more lipids to be integrated into membrane lipid rafts, activating membrane receptor tyrosine kinases and resulting in oncogenic signaling pathways [98]. Therefore, fatty acid synthase is associated with poor prognosis in patients with cancer [99]. It has been associated with clinically more aggressive cancers; in stage I breast cancer, there was a four-fold increase in mortality risk associated with the expression of fatty acid synthase in two studies [100]. FASN is correlated with peritumoral lymphatic vessel invasion and inversely correlated with breast cancer survival [101]. 

Fatty acid synthase is an attractive therapeutic target because it is expressed at low or undetectable levels in normal tissues because fatty acids are supplied by diet [100]. In contrast, since FASN is overexpressed in malignant cells, a FASN inhibitor would target the cancerous cells while leaving the normal cells unaffected [100]. It is also restricted solely to fatty-acid synthesis, unlike the other lipogenic enzymes. For example, acetyl-CoA carboxylase is the rate-limiting enzyme of fatty-acid synthesis but it is also widely distributed in muscle [100]. In colon cancer cells, inhibition of fatty acid synthase inhibited S-phase progression and DNA replication [100]. 

Preclinical evaluation of novel FASN inhibitors in primary colorectal cancer cells (CRCs) and a patient-derived xenograft model of colorectal cancer showed that anti-tumor activity was primary due to a significant decrease in the activation of Akt and Erk1/2 oncogenic pathways in CRCs [102]. Treatment with a FASN inhibitor led to the arrest of cell growth and apoptosis of breast tumor cells, further reinforcing the role of FASN in tumorigenesis [101]. FASN inhibition does this by upregulating pro-apoptotic genes BCL2 Interacting Protein 3 (BNIP3) and tumor necrosis factor related apoptosis-inducing ligand (TRAIL) [101]. BNIP3 induces apoptosis by mitochondrial dysfunction [101]. TRAIL has been found to be inversely correlated with FASN expression, which demonstrates that TRAIL is a component of the apoptotic pathway caused by FASN inhibition [101]. FASN can be inhibited using FASN small interfering RNA (siRNA). FASN inhibition by siRNA increases ceramide synthesis, which upregulates BNIP3 and TRAIL [101].

Cerulenin is another inhibitor of fatty acid synthase, and treatment demonstrated a cytotoxic property that was proportional to fatty acid synthase [100]. In other words, Cerulenin was selectively toxic and induced apoptosis in cancer cells but not normal cells in vitro [100]. Cerulenin is, however chemically unstable and therefore a more stable inhibitor of fatty acid synthase is needed as an anticancer agent [100].

**Figure 6:**
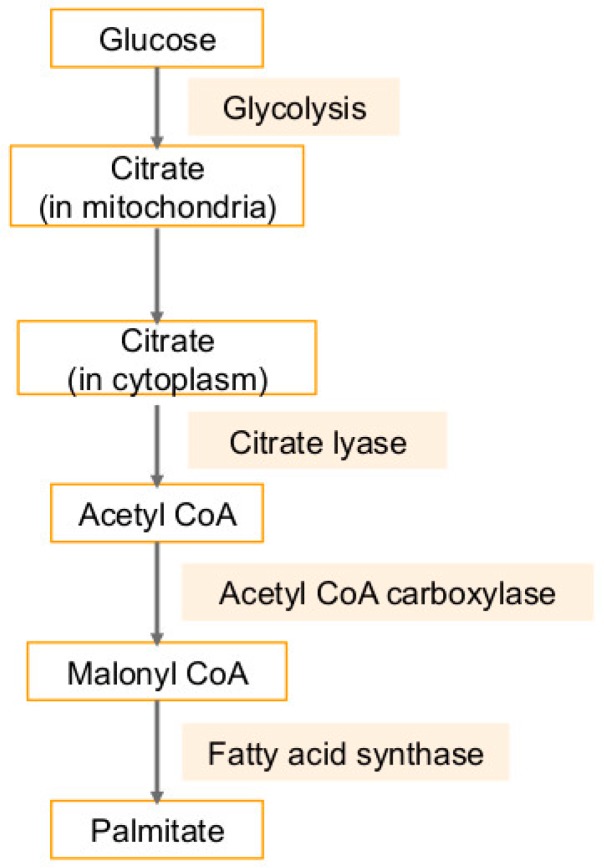
Fatty acid deregulation in pathogenesis of human cancer: A key lipogenic enzyme in fatty acid is fatty acid synthase (FASN), which can be inhibited to downregulate fatty acid synthase to inhibit DNA replication and arrest cell growth.

Hepatitis B interferes with lipid metabolism as well [103]. Fatty acid synthase is upregulated in HBV infected cells, and many studies have shown that HBV promotes the synthesis of fatty acids and cholesterol [103]. Lipid metabolism is altered during EBV infection as well [8]. Fatty acid synthase expression is increased in infected cells to engage de novo synthesis of palmitate, instead of relying on dietary fatty acids as healthy cells do [8] (Figure 6). This is confirmed by the findings that tumorigenesis in EBV infected cells driven by fatty acid synthase can be blocked by using fatty acid synthase inhibitors [8]. Moreover, treatment of infected cells with a fatty acid synthase inhibitor reduces cell survival [12]. Inhibition of key enzymes such as acetyl-CoA carboxylase also leads to apoptosis in infected cells [12]. EBV immediate-early (IE) protein BRLF1 activates expression of the host fatty acid synthase through a p38 stress mitogen-activated protein kinase and induces a lytic form of EBV replication [104]. 

KSHV infection also increases peroxisome biogenesis, and the proteins involved in the peroxisomal metabolism of very long chain fatty acids are critical for the survival of infected cells [105]. Reactivation is induced by short chain fatty acids, and angiogenesis is promoted by the vitamin D receptor pathway and increased cholesteryl ester synthesis [12].

## 5. Arachidonic Acid Pathway Metabolites in Cancers and Viral Infection-Associated Cancers

**Figure 7:**
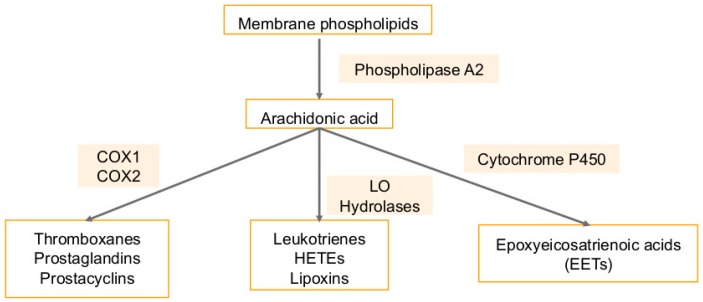
Arachidonic acid cascade: Arachidonic acid is a fatty acid that is freed from cellular membranes by phospholipase A2. It can then be metabolized to prostanoids such as thromboxanes (TXA2), prostacyclin (PGI2) and prostaglandins PGD_2_, PGE_2_, and PGF2a through the cyclooxygenase (COX) pathway. The lipoxygenase (LO) pathway along with hydrolases converts arachidonic acid into leukotrienes, hydroxyeicosatetraenoic acids (HETEs), and lipoxins (LXA4 and LXB4). Lastly, the cytochrome P450 monooxygenase pathway converts arachidonic acid into epoxyeicosatrienoic acids (EETs).

Many cancers have been shown to have aberrant metabolism of arachidonic acid [106,107,108,109,110]. Arachidonic acid is a fatty acid found in the cellular membrane [106] (Figure 7). It is metabolized to eicosanoids through 3 pathways: the cytochrome P450 monooxygenase (ω-hydroxylases and epoxygenases), the cyclooxygenases (COX-1 and COX-2), and the lipoxygenase (5-LO, 12-LO, 15-LOa, 15-LOb) pathways [106]. Metabolism by these pathways generates eicosanoids, such as leukotrienes and prostaglandins, which are pro-inflammatory and promote tumor growth [106,107,108,109,110] (Figure 7). Besides these eicosanoids are the epoxyeicosatrienoic acid (EETs), which are generated via the conversion of arachidonic acid by CYP epoxygenases and are mainly metabolized by soluble epoxide hydrolase (sEH). EETs, lipid signaling molecules are autocrine and paracrine mediators of cell proliferation, migration, inflammation, and angiogenesis in several tissues [111,112]. Pro-inflammatory eicosanoids are highly expressed in cancer cells and can promote tumor progression by inducing the secretion of growth factors by epithelial cells, inducing angiogenesis by binding receptors on stromal cells, regulating apoptosis, cell migration and proliferation by activating receptors on tumor epithelial cells and promoting a microenvironment that supports tumor growth [106]. 

The 5-LO expression is promoted by pro-inflammatory stimuli, and it is constitutively expressed in cancers including breast, prostate, lung, colon, and esophagus [106] (Figure 7). Inhibition of this pathway may reduce tumor growth and metastases, and have shown this in mouse models of human breast, esophageal, colon, and skin cancer [106,107,108,109,110]. The COX enzyme has two isoforms: COX1, which is ubiquitously expressed, and COX2, which is inducible [113] (Figure 7). COX2 is highly expressed during tumor formation and in areas of inflammation, and increased expression is associated with decreased rates of survival among patients with mesothelioma, breast, prostate, pancreas, liver, stomach, lung, and esophagus cancers [106,107,108,109,110] (Figure 7). Mammary angiogenesis and tumorigenesis is reduced in rodent models when COX2 is knocked out [113].

Both COX enzymes catalyze the ultimate production of prostaglandin E_2_ (PGE_2_), which upregulates the production of aromatase in fat cells and therefore more estrogen production, which promotes tumor cell proliferation [106]. PGE_2_ also upregulates NFkB activity, which is involved in inhibiting apoptosis and upregulates anti-apoptotic protein Bcl2 via Ras-MAPK signaling [106]. PGE_2_ has immunosuppressive effects as well: it upregulates immunosuppressive TH2 cytokines while downregulating anti-tumor TH1 cytokines [106]. It suppresses natural killer cells, which have anti-tumor activity, by inhibiting CD8+T activity [106]. A negative correlation has been shown between prostaglandin levels in breast tumors and survival [114].

PGE_2_ and leukotriene B4 (LTB4) promote angiogenesis by inducing vascular endothelial growth factor (VEGF), fibroblast growth factor 2 (FGF2), and chemokines CCL2 and CXCL1 [106]. They also shift the normal tissue microenvironment to one that supports tumor growth by exacerbating inflammation by recruiting more leukocytes into the tissue from the circulation [106].

Non-steroidal anti-inflammatory drugs (NSAIDs) inhibit COX and therefore have anti-inflammatory effects. These have been reported to reduce the risk of tumors in breast, lung, colon, and prostate cancers [106,107,108,109,110]. However, prolonged use of NSAIDs has cardiovascular and gastrointestinal side effects [106]. One way to avoid these may be to use antagonists of PGE_2_ receptors to inhibit the growth of tumors, which has been shown to have an effect in colon, lung, esophageal, and breast cancer animal models [106]. The cyclooxygenase-2-prostaglandin E_2_-eicosanoid receptor inflammatory axis has been demonstrated to play a key role in KSHV associated malignancies [115]. KSHV can activate parts of the lipoxygenase pathway such as leukotrienes and 5-lipoxygenase to aid in initial infection [12,116]. 

Concurrent inhibition of COX-2 and soluble epoxide hydrolase (sEH) using PTUPB, an orally bioavailable COX-2/sEH dual inhibitor results in antitumor, anti-angiogenic activity and has organ-protective effects [117]. PTUPB has the potential for future combination chemotherapy partner for cisplatin [117].

## 6. Use of Statins in Cancers, Viral Infections and Associated Cancers

Statins are competitive inhibitors of 3-hydroxy-3-methyl-glutaryl-coenzyme A reductase (HMGCR) and are referred to as cholesterol-lowering drugs as these regulate the rate-limiting step of the cholesterol synthesis pathway [118,119,120]. Statins target the MVA pathway by inhibiting HMGCR and reducing the levels of mevalonate and ultimately reducing plasma cholesterol [121] (Figure 3). HMGCR regulates the mevalonate pathway, and when upregulated, promotes tumor growth in breast cancer cells [122]. High levels of HMGCR are correlated with poor survival outcomes [122]. It has also been shown that HMG CoA reductase can induce growth in breast epithelial cells, independent of Anchorage [35]. Statins, therefore, reduce metastatic potentials and improve the survival rates in metastatic breast cancer mouse models [122,123]. Statins induce apoptosis of many cancer cell types and block pathways driving cell division in several types of leukemias and lymphomas [118,119,120]. Furthermore, an inverse correlation between cancer risk and statin use has been shown [124]. Studies have shown that statins reduce the incidence of breast cancer, colorectal cancer, prostate cancer, and cholangiocarcinoma [79]. Statins have been associated with lower risk of gastric cancer (due to weakening H. pylori infection), reduced hepatocellular carcinoma risk, and lower risk of melanoma [123,125,126]. By suppressing the mevalonate pathway, statins inhibit cell proliferation, trigger apoptotic parameters, and are powerful anti-inflammatory agents [121]. Another effect of blocking the mevalonate pathway is the regulation of the production of geranylgeranyl and farnesyl pyrophosphates, which modify the Rho and Ras -GTPases [127]. Rho proteins are associated with the invasive and proliferative properties of cancer cells [127]. Statins suppress the expression of matrix metalloproteinases (MMPs) and very late antigens (VLAs), and subsequently inhibit tumor growth and spontaneous metastasis [123]. They also induce cell apoptosis by inhibiting PI3K/Akt signaling [127,128] and produce anti-proliferative effects on lung, liver, colorectal, and prostate tumors [124]. Statins also modulate MAPK and CDK2, which reduce the expression of p21 and p27 cyclin kinase inhibitors, which regulate proliferation and apoptosis of tumor cells [127].

**Figure 8:**
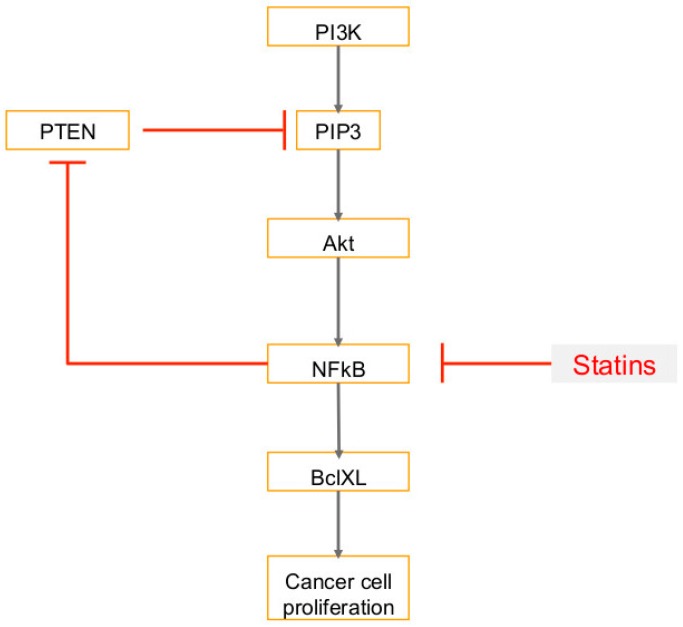
Statin therapy and downstream consequences: Statins block transcription of NFkB. This decreases expression of anti-apoptotic BclXL and inhibits cancer cell proliferation. Inhibition of NFkB also increases expression of pro-apoptotic phosphatase and tensin homolog (PTEN). PTEN inhibits PI3 kinase, and therefore also inhibits the target of PI3 kinase, Akt kinase, which are both associated with the anti-apoptotic properties of cancer cells. T-bars in red denote inhibition.

Studies in rodents show that lipophilic statins such as simvastatin, lovastatin, and fluvastatin have a protective effect on the growth of several tumor types by decreasing mevalonate synthesis [88]. Lovastatin arrests the cell cycle at the G1 phase by inducing cell cycle inhibitors p21 and p27, and decreases transition to the S and G2/M phases [83]. Lovastatin treated breast cancer cells show higher levels of caspase activity, which is consistent with apoptosis initiation [83]. Furthermore, cytochrome c was released by a reduction in the mitochondrial membrane potential and Bax translocation into the mitochondria [83]. Fluvastatin increases the rate of apoptosis and reduces breast tumor proliferation [129]. Simvastatin in particular has been shown to reduce the risk of several different cancer types, including breast cancer [125]. In mice that were treated with simvastatin, tumor growth and proliferation were inhibited and reduced [127]. Simvastatin is also associated with a reduced risk of breast cancer recurrence [4]. In bile duct cancer cells, simvastatin suppresses cell proliferation by inducing G1 phase cell cycle arrest [130]. It also activates caspase-3, downregulates Bcl-2 expression, and enhances Bax expression, which consequently induces apoptosis in bile duct cancer cells [130]. In prostate cancer, simvastatin suppresses metastasis by inhibiting TGF-β1, which promotes tumor progression and is associated with poor outcomes [131]. Statins were reported to prime cancer cells for apoptosis and worked in synergy with an inhibitor of BCL2 called venetoclax, and demonstrated better clinical responses compared to venetoclax alone [118,119,120]. In breast cancer cells, simvastatin affects several signal transduction pathways and changes expression of Akt, NFkB, BclXL, and PTEN [127]. Simvastatin reduces cell viability through reduction of raft formation, which consequently downregulates survival kinase Akt [82]. Akt promotes cell survival and inactivates pro-apoptotic proteins, and is therefore characteristic of malignant tumors [82]. Simvastatin blocks phosphorylation of Akt, which inhibits Akt activation [127]. Simvastatin inhibits NFkB transcriptional activity, which subsequently decreases expression of anti-apoptotic protein BclXL and increases levels of phosphatase and tensin homolog (PTEN), a tumor suppressor protein [127] (Figure 8). Akt regulates many transcription factors, including NFkB, which is constitutively active in breast cancer cells [127]. NFkB induces expression of several anti-apoptotic proteins including BclXL [127]. Breast cancer cells have higher levels of BclXL, and simvastatin inhibits its transcription by targeting NFkB [127]. In breast cancer cells, a mutation or deletion in PTEN increases levels of PI3 kinase product PIP3 [127], which consequently upregulates Akt activity [127]. Activation of phosphatidylinositol 3 kinase (PI3 kinase) and its target Akt kinase is associated with the anti-apoptotic properties of cancer cells [127]. PI3 kinase is negatively regulated by phosphatase and tensin homolog (PTEN), a tumor suppressor protein [127]. PTEN controls the levels of the product of PI3 kinase: PI 3, 4, 5-triphosphate (PIP3), which activates Akt kinase [127]. Simvastatin increases expression of PTEN to reduce activation of Akt by targeting NF-κB to inhibit its repression of PTEN expression and therefore inhibit breast cancer cell proliferation [127]. Simvastatin thus increases transcription of phosphatase and tensin homolog (PTEN), which suppresses the oncogenic phosphatidylinositol-3-kinase (PI3K) pathway and reduces expression of the anti-apoptotic protein bcl-XL [129].

Two other pathways simvastatin uses to suppress cell growth and induce apoptosis are the MAPK/ERK pathway and the JNK/CHOP/DR5 pathway [132]. Simvastatin suppresses tumor growth by suppressing the MAPK/ERK pathway, which promotes cancer cell survival and metastasis [132]. Simvastatin also activates the JNK/CHOP/DR5 pathway, which induces apoptosis in breast cancer cells [129]. Simvastatin has been reported to inhibit the activity of HBV minichromosome maintenance (MCM) 7 complex, an important host factor aiding virus genome replication in host cells by increasing the phosphorylation of eIF2α, which is mediated by the liver kinase B1 (LKB1)-AMP-activated protein kinase (AMPK) signaling pathway [133]. Statin use may have potential protective effects and reduce the risk for hepatocellular carcinoma (HCC) in HBV-infected patients in a dose-dependent manner [134]. 

Treatment with statins, which downregulate the mevalonate pathway, has been shown to block hepatitis C virus [89]. Statin treatment affected cell cycle, apoptosis and alternative splicing genes between the native (B-lymphocytes) and EBV transformed cells human lymphoblastoid cells (LCLs) [135]. Simvastatin has been shown to delay the development of EBV-lymphomas in severe combined immunodeficiency mice models and could be considered for the treatment of EBV-lymphomas [136]. Anti-HCV effects or reduced HCV replication via Ceestatin, a drug that binds to 3-hydroxy-3-methylglutaryl-coenzyme A (HMG-CoA) synthase and irreversibly inhibits HMG-CoA synthase in a dose-dependent manner are reversed by addition of HMG-CoA, mevalonic acid, or geranylgeraniol [137]. Side effects of statins include acute renal failure, myopathy, myoglobinuria, and hepatotoxicity [138]. Also, increased risk of diabetes mellitus and muscle pain has been observed [79]. Recent studies have found an increased risk of cancer after use of pravastatin, which caused an increase of mevalonate synthesis in extrahepatic tissues and thus promoted the growth of breast cancer cells. No such association has been found with use of lipophilic statins such as simvastatin, lovastatin or fluvastatin [88]. 

## 7. Use of Fibrates (PPARα Agonist) in Cancers, Viral Infections and Associated Cancers

Fibrates are an effective and safe group of hypolipidemic drugs, frequently used to treat patients with atherogenic dyslipidemia and have shown tremendous potential in cardiovascular outcomes [139,140,141]. Fibrates also have benefits in carbohydrate metabolism, adipokines levels, thrombosis, atherosclerosis (hardening of arteries), heart attack, stroke and inflammation [139,140,141]. Fibrates such as fenofibrate can inhibit triglyceride (TG) synthesis via reducing the availability of free fatty acids by promoting β-oxidation and also has anticancer agent potential [139,140,141]. Fibrates are well-known ligands of peroxisome proliferator-activated receptor α (PPARα). PPARs are ligand-activated transcription factors that modulate lipid metabolism and are involved in transcription of genes that regulate cell proliferation [24,98]. This family of nuclear receptors is expressed in tumors cells as well as in tumor endothelium [142]. PPARα is a particularly important transcriptional regulator of inflammatory and metabolic processes, which means its agonists can be used to treat dyslipidemia [98]. Functional activity of PPAR is maintained by histone deacetylase (HDAC), co-repressors/co-activators, retinoid X receptors (RXRs), RNA polymerase II, histone acetyltransferases (HATs), phosphorylation and dephosphorylation [139,140,141].

PPARα ligands suppress cancer cell growth in several lines, including breast, colon, and skin [142]. They also suppress the metastasis of melanoma cells [142]. PPARα ligands can induce apoptosis of endothelial cells and inhibit proliferation and migration [142]. PPARα ligands produce anti-inflammatory effects by inhibiting NOS, COX-2, and tumor necrosis factor TNFα, and reducing inflammation in tumors may be associated with inhibition of cell growth and improved prognosis [142]. Moreover, PPARα deficiency suppresses angiogenesis by producing excess thrombospondin (TSP-1) and also prevents tumor growth [142]. Given these reasons, PPARα ligands could potentially be tumor-preventing agents. Fibrates in particular target the MVA pathway by inhibiting acetoacetyl coenzyme A [143,144] (Figure 3). 

Clofibrate inhibits cell growth by changing the levels of checkpoint kinases, cell cycle inhibitors, and tumor suppressors [98]. In breast cancer cells, higher levels of PPARα are found when compared to HMEC cells [98]. In these cells, Clofibrate reduces the amount of fatty acid synthase, inhibits growth, and reduces survival kinases [98]. In breast cancer cells, COX-2 expression promotes cell adhesion and migration, which in turn accelerates cancer progression [98]. Clofibrate, in turn, downregulates COX-2 as well as 5-LO inflammatory pathway components [98]. In rodents, the use of fibrates for prolonged periods of time can cause peroxisome proliferation, which leads to hepatomegaly and tumor formation. As this has not been demonstrated in humans, a low dose of fibrates for a short duration of time can be used [98]. PPAR agonists have also been associated with weight gain [24]. 

## 8. Other Lipid Metabolites and Pathways in Cancer and Viral Infections

Beside the pathways and metabolites mentioned here, there are many other very important lipid pathways mediating enzymes and metabolites. Gangliosides along with other glycosphingolipids, phospholipids, and cholesterol in glycolipid-enriched microdomains present at the outer leaflet of the plasma membrane interact with signaling molecules including receptor tyrosine kinases and signal transducers and contribute to activation of cell signaling, increasing cell proliferation and migration, as well as tumor growth [145].

Phospholipase D (isoenzymes PLD1 and PLD2) catalyzes the hydrolysis of cell membrane phospholipids and plays the role in several cancers and infectious diseases [146]. PLD inhibitors have been proposed as novel compounds for the treatment of cancers, neurodegenerative disorders, and viral infections [147]. PLD activity has been linked with promoting survival and metastatic phenotype of malignant prostate cancer cells [148] and PLD inhibitors reduce human prostate cancer cell proliferation and colony formation [149]. HPV infection, the key risk factor for the development and progression of cervical cancer utilizes PLD and Phosphatidic acid (PA) and regulate the PI3K/AKT/mTOR pathway [150].

ATP citrate lyase (ACLY) is involved in lipid during membrane biogenesis (fatty acid synthesis) linked with aerobic glycolysis. ACLY catalyzes the conversion of citrate to oxaloacetic acid (OAA) and acetyl-CoA [151]. Acetyl-CoA is the acetyl donor for lysine acetylation that links metabolism, signaling, and epigenetics. ACLY overexpression and activation increase metabolic activity in proliferating cells via activation of Akt signaling in glioblastoma, colorectal cancer, breast cancer, non-small cell lung cancer, and hepatocellular carcinoma etc. [152]. There are varying reports on the effect of ACLY depletion on cancer cells. Few suggest that genetic deletion of ACLY does not kill cells but these cells start proliferating at the impaired rate by utilizing exogenous acetate for de novo lipogenesis and histone acetylation [153]. There are studies in breast cancer, which suggest that depletion of ACLY suppressed breast tumor growth and progression [151]. Low molecular weight cleaved form of cyclin E, a powerful independent predictor of survival in women with progressed breast cancer, was shown to interact with ACLY and promote aberrant lipid metabolism pathways in breast cancer tumorigenesis [154]. ACLY has been implicated in integrin signaling in glioblastoma leading to cell adhesion and migration required for its progression and metastasis [155]. ACLY activation during hepatitis B virus (HBV) has been reported to be involved in the disturbed lipid metabolism and proliferation of hepatocytes [156]. ACSS2-mediated acetyl-CoA synthesis from acetate has been shown to take over ACLY functions during HCMV infection, which has been associated with malignancies including colon cancer, malignant glioma, prostate carcinoma, and breast [157,158,159,160].

## 9. Summary

Diet and obesity play an important role in breast, prostate, gastric, lung and skin cancer development, and increased lipogenesis has been observed in tumor cells of virally infected hosts [23,161]. This may be caused by disruptions in the same signaling pathways responsible for the oncogenic transformation of cancer cells [161]. The challenge, therefore, is to discover novel, non-toxic therapies that target these essential steps in the development of lipid/obesity-associated cancers. One such pathway, the mevalonate pathway, can be dysregulated in cancer cells and lead to poor prognosis in cancer patients, as cholesterol has been shown to accelerate tumor formation [23,87]. Statins target this pathway by inhibiting HMGCR, which reduces plasma cholesterol and has to been shown to ultimately reduce cancer risk [121,124]. Another possible therapeutic target is fatty acid synthase, which is expressed at high levels in cancer cells and leads to more lipogenesis and oncogenic signaling pathways [98]. Treatment with a FASN inhibitor increases apoptosis and decreases cell growth in cancer cells [101]. PPARs are another modulator of lipid metabolism and regulate cell proliferation [24,98]. PPARα ligands, fibrates, suppress breast cancer cell growth [98]. Lastly, abnormal metabolism of arachidonic acid in cancer cells leads to high levels of pro-inflammatory eicosanoids, which promote tumor progression [106]. NSAIDs inhibit the inflammatory COX pathway and have been reported to reduce the risk of cancer [106]. Since viral infection associated cancers also heavily utilize lipid metabolism for their growth and progression, therefore there could be common targets which could be tested to combat viral cancers. Numerous studies discuss and show promising results targets for NSAIDs [116,162,163,164,165,166,167,168], mTOR pathway regulators [169,170,171,172], and FASN inhibitors [75] in viral infection associated cancers but additional in vivo studies need to be done to fully exploit these pathways. These studies support the further development of statins, FASN inhibitors, fibrates, and NSAIDs as potential anticancer agents.

## Abbreviations

KSHV	Kaposi’s sarcoma-associated herpesvirus
MCPyV	Merkel cell polyomavirus—a polyoma virus
MCC	Merkel cell carcinoma
EBV	Epstein–Barr virus
HPV	human papilloma virus
HTLV	human T lymphotropic virus
cART	combination of anti-retroviral therapy
IGF-1	insulin-like growth factor 1
SHBG	sex-hormone binding globulin
BARD1	BRCA1-associated RING domain protein 1
TNFα	tumor necrosis factor-alpha
MAPK	mitogen activated protein kinase
HMEC	human mammary epithelial cells
OPG	osteoprotegerin
SREBF2	Sterol regulatory element-binding protein transcription factor 2
LXR	Liver x receptors
PPARs	Peroxisome proliferator—activated receptors
HCC	hepatocellular carcinoma
LKB1	liver kinase B1
HMG-CoA synthase	3-hydroxy-3-methylglutaryl-coenzyme A synthase
MCM	minichromosome maintenance
PTEN	phosphatase and tensin homolog
LTB4	leukotriene B4
MMPs	matrix metalloproteinases
PLD	Phospholipase D
ACLY	ATP citrate lyase
VEGF	vascular endothelial growth factor
FGF2	fibroblast growth factor 2
NSAIDs	Non-steroidal anti-inflammatory drugs
EETs	epoxyeicosatrienoic acid
STAR	steroidogenic acute regulatory
FASN	fatty acid synthase
ABCA1	ATP-binding cassette transporter
ACSL1	Acyl-CoA Synthetase Long Chain Family Member 1
AGPAT1	1-Acylglycerol-3-Phosphate O-Acyltransferase 1
SCD	Stearoyl-CoA desaturase (Δ-9-desaturase)
RAC	Rectal adenocarcinoma
PDAC	Pancreatic ductal adenocarcinoma
MUFAs	mono-unsaturated fatty acids
SCFA	Short-chain fatty acids
PI3K	phosphatidylinositol 3-kinase
HIFs	hypoxia inducible factors
ESCRT	endosomal sorting complexes required for transport
ELOVL6	fatty acid elongase
CPT	Carnitine palmitoyltransferase.
